# ICP-MS-based characterization of inorganic nanoparticles—sample preparation and off-line fractionation strategies

**DOI:** 10.1007/s00216-013-7480-2

**Published:** 2013-12-01

**Authors:** Anne-Lena Fabricius, Lars Duester, Björn Meermann, Thomas A. Ternes

**Affiliations:** Department G2—Aquatic Chemistry, Federal Institute of Hydrology, Am Mainzer Tor 1, 56068 Koblenz, Germany

**Keywords:** Nanoparticle quantification, Total concentration analyses, Off-line fractionation, Sample preparation, Dissolved fraction, ICP-MS

## Abstract

Validated and easily applicable analytical tools are required to develop and implement regulatory frameworks and an appropriate risk assessment for engineered nanoparticles (ENPs). Concerning metal-based ENPs, two main aspects are the quantification of the absolute mass concentration and of the “dissolved” fraction in, e.g., (eco)toxicity and environmental studies. To provide information on preparative aspects and on potential uncertainties, preferably simple off-line methods were compared to determine (1) the total concentration of suspensions of five metal-based ENP materials (Ag, TiO_2_, CeO_2_, ZnO, and Au; two sizes), and (2) six methods to quantify the “dissolved” fraction of an Ag ENP suspension. Focusing on inductively coupled plasma–mass spectrometry, the total concentration of the ENP suspensions was determined by direct measurement, after acidification and after microwave-assisted digestion. Except for Au 10 nm, the total concentrations determined by direct measurements were clearly lower than those measured after digestion (between 61.1 % for Au 200 nm and 93.7 % for ZnO). In general, acidified suspensions delivered better recoveries from 89.3 % (ZnO) to 99.3 % (Ag). For the quantification of dissolved fractions two filtration methods (ultrafiltration and tangential flow filtration), centrifugation and ion selective electrode were mainly appropriate with certain limitations, while dialysis and cloud point extraction cannot be recommended. With respect to precision, time consumption, applicability, as well as to economic demands, ultrafiltration in combination with microwave digestion was identified as best practice.

**Electronic supplementary material:**

The online version of this article (doi:10.1007/s00216-013-7480-2) contains supplementary material, which is available to authorized users.

**Figure:**
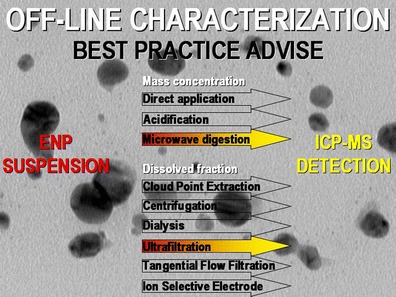
A Multi-method approach to identify best practice for ICP-MS based off-line characterization of ENP suspensions.

## Introduction

Due to an increased use of engineered nanoparticles (ENPs) in a variety of products and applications, the exposure of workers and consumers as well as the release into the environment has to be expected. To ensure the safety of the different ENPs and to reduce environmental and (eco)toxicological impacts, an appropriate risk assessment and the development of adequate regulatory frameworks are required. This demands not only for a comprehensive number of scientific studies but also for the availability of validated and easily applicable analytical methods [1–3]. These tools should also be implementable by non-“nano”-specialized laboratories (e.g., in (eco)toxicological and environmental research or administrative services). Until now, the detection, characterization, and quantification of ENPs, especially in different matrices, are still challenging tasks and the analytical methods required to provide sufficiently reliable data are most often laborious, expensive, and/or demand specialized and trained operators. Standardized protocols developed for the analysis of chemicals are mostly not directly applicable to ENP suspensions since they do not account for physicochemical parameters which are important for nanoparticle characterization (like size, shape, agglomeration/aggregation state, or surface area). Furthermore, many techniques are only suitable for specific samples or sample matrices or are limited to a certain range of, e.g., concentration or size [4–6]. Since only a few size-, but not mass- and number- concentration certified nanoparticle reference materials are available (e.g., NIST reference materials 8011-8013 [7], ERM-FD100 and ERM-FD304 [8], or BAM-N001 [9]), the validation of analytical methods for ENPs is challenging and often multi-method approaches are required to provide reliable data as well as to assess and control the limitations of different techniques [6, 10, 11]. Beside this, it has to be taken into account that the properties of ENPs can differ strongly among each other and from their (chemically identical) bulk material and may vary over time or in dependence on the surrounding matrix [12–14]. Some of the most important questions regarding the analysis of nanomaterials are connected to fractionation, since the particle size and the percentage of the dissolved fractions may have a strong impact on toxicity [15–18] and fate [13, 19, 20]. It is hence crucial to distinguish between the particulate and the dissolved fraction to gain an understanding on the fate and transport characteristics of the particles and ionic forms and their possible (independent or synergistic) environmental and (eco)toxicological impacts [1]. Regarding the determination of the dissolved fraction of ENP suspensions, several aspects, including the properties of the particles (e.g., size, shape, aggregation state, surface characteristics, coatings) [21–23] and of the surrounding matrix (pH value, temperature, ionic strength) [22, 24, 25] as well as the concentration and the intensity/duration of the dissolution process, have to be taken into account [26, 27]. Moreover, it has to be considered that the percentage of the dissolved fraction can, but does not necessarily, reach a stable status [28, 29] and that also a complete dissolution is possible [22, 25, 28]. Commonly applied methods to separate the dissolved fraction from the particles are, e.g., centrifugal ultrafiltration [16, 21, 24, 25, 30], (ultra)centrifugation [5, 28], dialysis [16, 26], or the detection of silver ions by ion selective electrodes [30–32]. Irrespective of the separation method applied, a precise quantification of the total mass concentrations of the different fractions is indispensable, not at least because the actual dose metric-based risk assessment demands for an exact determination to enable a comparison with the results of former exposure or (eco)toxicological studies [1–3]. As a standard technique in elemental analysis, inductively coupled plasma–mass spectrometry (ICP-MS) has become one of the most commonly used tools for the determination of the total concentration of inorganic nanoparticle suspensions [32, 33]. One increasingly applied technique to quantify directly the total concentration of the particulate and the dissolved fraction of ENP suspensions is single particle ICP-MS (SP-ICP-MS). Even though this method provides a powerful tool for the characterization of most metal-based nanoparticles, it is a rather sophisticated method that demands well-educated and trained operators since several special considerations regarding, e.g., the instrumental parameters (e.g., dwell time or detector dead time), the sample characteristics (e.g., preferably monodisperse suspensions and spherical and solid particles), and handling (e.g., volume and concentration of the sample introduced) or the interpretation of the data have to be taken into consideration [34–36].

In terms of classical measurement approaches (based on steady state signals), it is still questionable if mass concentrations can be determined properly by a direct application of suspensions via the nebulizer and the spray chamber. Even though some publications point out that ENP suspensions require a digestion procedure prior to the measurements [4, 32], the actual biases for directly measured ENP suspensions were (to the best of the authors' knowledge) not addressed in detail yet. However, for daily routine analysis, fast and simple sample preparation protocols are needed, and in case of fractionation techniques coupled on-line to ICP-MS (e.g., field flow fractionation, hydrodynamic chromatography, or size-exclusion chromatography), a digestion of the sample prior to quantification is impossible.

This study aims to investigate (1) if and which sample preparation procedure is required prior to ICP-MS measurements to quantify precisely the total concentration of some of the most commonly used and discussed nanoparticle suspensions (Ag, TiO_2_, CeO_2_, ZnO, Au) [37, 38]. Therefore, three sample preparation approaches were compared, including microwave-assisted digestion, acidification of the suspensions, as well as direct measurement via ICP-MS without further preparation. In addition to the ICP-MS-based analysis, a gravimetric approach was carried out. Beside the determination of the total concentration, (2) the question how the dissolved fraction of an ENP suspension can be determined properly was addressed. Therefore, a quantitative multi-method approach was undertaken to elucidate the advantages and limitations of different, preferably easily implementable approaches. Using a silver ENP suspension, five off-line fractionation methods (two filtration techniques, dialysis, ultracentrifugation, and cloud point extraction) were compared to determine the “dissolved” fraction, supplemented by measurements with an ion-selective electrode (ionic silver). Special attention was put on the uncertainties of the methodologies and their limitations.

Focusing on the challenge to develop standardized and easily implementable analytical protocols for nanomaterials, the study presented delivers best practice advice. With this so far missing approach, the reliability of studies that include fractionation and total mass determination can be improved. The study supports the development of standard protocols for validated analyses of ENP suspensions suitable for everyday applications, which are requested in several directives. Hence, it supports the urgently needed process from “specialized nano analytics” to “customary nano analytics.”

## Experimental section

### Chemicals and materials

Acids; ICP-element standards of Ag, Ti, Ce, Zn, Au, and Ru (1 g/L); as well as hydrogen peroxide and sodium thiosulfate pentahydrate were purchased from Merck GmbH (Germany). Sodium nitrate (pro analysis grade) was obtained from Carl Roth (Germany). Hydrochloric (30 %) and sulfuric acid (96 %) as well as hydrogen peroxide (30 %) were at high purity grade (Suprapur); nitric acid (65 % *w*/*w*, for analysis) was sub-boiled (dst-1000, Savillex, USA). Ultrapure water (18.2 MΩ × cm) was produced using an Arium pro VF system (Sartorius AG, Germany). Prior to use, all vessels were rinsed >24 h with HNO_3_ (1.3 %).

### Nanoparticle suspensions

Eight different ENP suspensions were examined: The silver (Ag) nanoparticle suspension was obtained from RAS materials GmbH (Germany; AgPURE-W, suspended in 3–5 % ammonium nitrate solution), cerium dioxide (CeO_2_) by Nyacol Nanotechnologies Inc. (USA, 20 wt%, 3 % acetic acid). Titanium dioxide (TiO_2_, anatase) powder was kindly provided by Tronox (Germany). Stable, additive-free TiO_2_ suspensions were produced by a high-power-density ball milling procedure (for detailed information refer to Duester et al. [39]). Zinc oxide (ZnO) was purchased from Particular GmbH (Germany; suspended in sodium citrate solution ∼100 mg/L); gold (Au) ENP suspensions were obtained from Sigma-Aldrich Chemie GmbH (Germany, suspended in 100 mg/L sodium citrate solution, stabilized by proprietary surfactants not further described by the manufacturer). Further information provided by the manufacturer can be found in the Electronic Supplementary Material (Table S1). For the two-method comparisons conducted (sample preparation procedures and determination of the dissolved fraction), the stock suspensions were diluted to working suspensions (WS) of a mass concentration of approximately 1 to 10 mg/L (refer to the Electronic Supplementary Material, Table S1).

### General characterization of the ENP suspensions

#### Particle size distribution and zeta potential

Prior to the experiments, the particle size distribution of the ENP suspensions was determined by means of Dynamic Light Scattering (DLS; ZetaSizer NanoZS, Malvern Instruments GmbH, UK) and Nanoparticle Tracking Analysis (NTA; LM10, Nanosight ltd., UK). In case of the ZnO suspension, aggregation was observed (∼1,700 nm) and ultrasonication was applied to obtain reproducible particle size distributions. This was conducted using an ultrasonic homogenizer (GM 3100 HF-Generator, UW 3100 ultrasonic converter, equipped with a mycro-sonotrode MS 72; Bandelin electronic GmbH Co. KG, Germany) applying the following sonication program: 3 × 2 min, 40 W, in a pulse mode of 20 s energy/5 s pause. The other suspensions were stable over the experimental period and re-dispersion by ultrasonication was not necessary. Transmission electron microscopy (TEM; Philips EM-420, Philips, Netherlands) measurements were carried out at an acceleration voltage of 120 kV, equipped with a LaB6 cathode and a slow-scan CCD camera. Twenty-five microliters of the suspension was placed onto graphite-coated copper grids and dried over night prior to the measurements. Mean particle sizes were estimated on the basis of the TEM images measuring at least 57 particles per sample. Zeta potential measurements were carried out using a ZetaSizer NanoZS (Malvern instruments GmbH, UK).

### Total concentration

#### Sample preparation procedures

A comparison of different sample preparation procedures was conducted, including microwave-assisted digestion, acidification, and dilution in ultrapure water. For microwave-assisted digestion, 0.5 mL of the suspension was mixed with 1.4 mL of acid and 0.1 mL of an internal standard (Ru). Detailed information about the experimental setup (acids, concentrations, pH values of the WS, reference materials) is given in the Electronic Supplementary Material (Table S2). Using a microwave system (turboWave, MLS GmbH, Germany), suspensions were digested in a two-step program of a temperature ramp of 60 min to 240 °C (800 W), followed by an irradiation of 30 min at 240 °C (800 W). ZnO and Au ENPs were digested in a mixture of HNO_3_ (1.1 mL ∼65 %) and HCl (0.3 mL ∼30 %), for Ag ENPs HNO_3_ (1.4 mL ∼65 %), for CeO_2_ a mixture of HNO_3_ (1.2 mL ∼65 %) and H_2_O_2_ (0.2 mL ∼30 %) was used. Digestion of TiO_2_ ENPs was conducted in H_2_SO_4_ (1.4 mL ∼96 %). The digested samples were diluted to 10 mL using ultrapure water. Previous to each experiment, blank values of the microwave vessels were determined, replacing the sample volume of the ENP suspensions (0.5 mL) by ultrapure water. At least six replicates of the ENP suspensions were investigated, whereat, for method validation purposes, in parallel to three replicates of the ENP suspension a blank sample (ultrapure water), a certified reference material (CRM) as well as an ICP-element standard (diluted to 1 mg/L) were analyzed. Experiments exhibiting that the expected values for CRM and/or ICP-element standard were biased >10 % were excluded from the evaluation. Equally, in cases where the IS added differed between the digested and the acidified samples >10 %, the results were waived. Digested suspensions were compared with acidified samples (same mixture but not digested) and directly measured nanoparticle suspensions, which were diluted in ultrapure water (instead of acid). Both sample preparations were performed at the same day of the measurements. The total elemental concentrations of the samples were determined by means of ICP-Quadrupole-MS (ICP-QMS; Agilent 7700 series, Agilent Technologies, Germany). The ICP-MS was equipped with a PEEK Mira Mist nebulizer (Burgener research, Canada) and a PFA inert sample introduction kit with a sapphire injector (inner diameter 2.5 mm, for Agilent 7700 series, Agilent Technologies, Germany). Measurements were conducted at a RF power of 1,550 W and a carrier gas flow of 1.17–1.18 L/min. Details about the isotopes analyzed, the measurement modi applied, as well as the reference materials used are given in the Electronic Supplementary Material (Table S2 and Table S3). Except for Au, the external calibration of ICP-QMS was matrix-matched. In case of Au, only a HCl matrix was used (to ensure the formation of stable gold–chloride complexes) since low concentrations (approx. <100 μg/L) of dissolved Au are instable. To verify the calibration procedure, CRMs were included in each measurement.

#### Comparison of ICP-MS and gravimetry

In addition to the ICP-MS-based analysis, a gravimetric approach was applied as an absolute and direct method. The results were compared to the concentrations determined upon microwave-assisted digestion of the stock suspensions (*n* >3), applying the approach described above. Since in case of Au differences between the results of the two approaches were observed, concentrations were additionally determined by means of graphite furnace–atomic absorption spectrometry (GF-AAS, vario 6, Analytic Jena AG, Germany). The spectrometer was equipped with transversely heated graphite tube with an integrated platform and a gold hollow cathode lamp (absorption line set to *λ* = 242.8 nm; slit width, 0.8 nm). After addition of 5 μL of a Pd/Mg(NO_3_) modifier, measurements were carried out at a pyrolysis temperature of 800 °C (10 s) and an atomization temperature of 1,950 °C (4 s). Instrument calibration was performed by external calibration via elemental standards; for quantification, signal-peak areas were determined upon integration (interval of 3.5 s).

For the gravimetric analysis, aluminum cups were annealed for at least 8 h at 450 °C by means of a muffle furnace (LE 6/11, B150, Nabertherm, Germany) until a constant weight was reached. Depending on the concentration expected (based on the producer information), a sample volume of 0.2 to 1.8 mL was pipetted into the cups and, again, repeatedly annealed to a constant weight. Weighing was conducted using a Sartorius M2P balance (Germany). To avoid biases by potential oxidation processes, combustion experiments were conducted in a nitrogen as well as atmospheric atmosphere. Only in case of silver the inert gas was required; for the other ENP suspensions, no differences were found between the results of the two treatments. To enable the comparison of the results measured by means of ICP-QMS and of those determined gravimetrically, the elemental concentrations were, in case of oxidic ENPs, converted into the concentrations of the oxides.

#### Precision of ICP-MS measurements

To investigate the influence of the particle size on the signal precision of the ICP-MS analysis, the relative standard deviations (RSD%; given by the ICP-MS software) of the measurements conducted were assessed (fivefold measurements). Furthermore, a direct application approach was undertaken, comparing suspensions of Au ENP of different sizes (10, 30, 80, 200 nm, diluted 1:1,000) as well as an ICP-element standard solution (100 ng/L). Measurements were carried out using a sector field ICP-MS (ICP-SF-MS, Element 2, Thermo Scientific, Germany) equipped with a μ-flow PFA-ST ES-2040 nebulizer (both from Elemental Scientific Inc., USA) and a 1.8-mm sapphire injector (Elemental Scientific Inc., USA). RF power was set to 1,500 W, carrier gas flow to 1.205 L/min.

### Dissolved fraction determination

#### Comparison of different off-line fractionation approaches

To investigate the suitability of different methods available for dissolved-fraction determination of ENP suspensions, five off-line fractionation experiments were conducted using a silver nanoparticle WS (AgPure, diluted 1:10,000), supplemented by measurements with an ion selective electrode (ISE; Ag/S 800, measurement range 10 μg/L–108 g/L, WTW, Germany) in connection to a MultiLine P4 Universal Meter (WTW, Germany). Focusing on preferably fast and easily applicable approaches, the fractionation methods considered were dialysis, centrifugation, ultrafiltration (UF), and tangential flow filtration (TFF). Additionally, cloud point extraction (CPE) was included, even though this method was originally developed to extract and concentrate nanoparticles rather than to quantify the dissolved fraction [31, 40, 41]. Parallel to the Ag ENP suspension, an ICP-MS single element standard (10 mg/L) and (for blank verification) ultrapure water were analyzed in triplicate. ENP suspensions were analyzed in (at least) six replicates. The ICP-silver standard was included to ensure the suitability of the methods for a quantitative analysis of the dissolved fraction and to estimate possible biases of the results caused by the procedure (e.g., losses due to interaction with the membranes). The percentage of “dissolved” silver (defined as <10 kDa molecular weight cutoff (MWCO) of the membranes) was calculated in relation to the total silver amount determined upon microwave-assisted digestion of the Ag ENP WS. Since the dissolution of silver in Ag ENP suspensions depends (among others) on the concentration of the suspension [25, 26], all experiments were carried out using a WS with an age of 2 to 5 weeks, after the equilibrium status was verified by ultrafiltration.

Cloud point extraction was carried out in accordance to Chao et al. [31]. In brief, 1 mL sample, 0.2 mL Triton-X 114 10 % *w*/*v* (diluted in ultrapure water; TR-X 114; Fisher Scientific GmbH, Germany, general purpose grade), and 0.1 mL Na_2_S_2_O_3_·5H_2_O (248.20 g/L) were diluted in 8.7 mL of acidified water (∼pH 2.9, adjusted by means of HNO_3_ addition) and incubated for 30–40 min at 40 °C. To accelerate the phase separation, samples were centrifuged for 10 min at 5,000 rpm (Sigma Laboratory Centrifuge 3 K30, Sigma Laborzentrifugen GmbH, Germany). Eight milliliters of the upper aqueous phase was sampled to determine the dissolved fraction.

For the centrifugation experiments, a volume of 12 mL was centrifuged (ultracentrifuge, Sorvall WX90, Thermo Scientific, Germany, swinging bucket rotor; TH-641) for >48 h at 41,000 rpm and 4 °C. The centrifugation time at a speed of 41,000 rpm (maximum of the rotor) for sedimentation of silver particles of approximately 50 nm (determined by DLS; see “Results and discussion” section) was estimated on the basis of the procedure described in detail by Griffith [42] and the information provided by the manufacturer of the centrifuge (further details are given in the Electronic Supplementary Material). To avoid redispersion of the nanoparticles, 1.1 mL of the supernatants was taken from the surface of the sample directly after centrifugation was finished. One milliliter was used to verify the efficiency of the method by NTA analysis by screening for particles remaining in the supernatant. To determine the total concentration of silver via ICP-QMS, 0.1 mL was used.

In case of the filtration methods (dialysis, UF and TFF), the “dissolved” fraction was defined by the MWCO of the membranes of 10 kDa, corresponding to approximately 1–2 nm. Dialysis was carried out on a multi-position magnetic stirrer (Variomag Telemodul 40S connected to a Variomag Electronicstirrer Telesystem, Thermo Scientific Germany) in 1 L bottles containing ultrapure water. Dialysis devices (Float-A-Lyzer G2, 8–10 kDa, Spectrum Laboratories Inc., USA) were filled with Ag ENP suspension or Ag solution (∼ 9 mL) and dialyzed for 48 h. To investigate the time-depended dissolution of Ag^+^ from Ag ENPs, additional samples were taken over a time period of 29 days (for time-dependant dissolution, refer to the Electronic Supplementary Material, Fig. S2). To determine the dissolved fraction, the concentration of silver in the surrounding water was measured via ICP-QMS. Ultrafiltration (Amicon Ultra Centrifugal filter units, MWCO 10 kDa, Merck Millipore, Germany) and TFF (Microkros Hollow Fiber Filter Module, 10 kDa, Spectrum Laboratories Inc., Netherlands) were carried out in accordance to the operating instructions of the supplier. In case of UF and TFF, a possible clogging of the membranes by ENPs was tested after the experiments by filtering a dissolved elemental standard.

ISE was calibrated in a concentration range from 100 μg/L to 100 mg/L in accordance to the instructions given in the manual of the electrode. To provide a constant ionic strength required for optimal ISE measurement conditions, 2 % NaNO_3_ solution (424.95 g/L) was added to each sample. The precision of the ISE was tested after calibration conducting a standard addition of a dissolved Ag ICP standard to the WS (1–50 mg/L). The calibration as well as the results of the standard addition are given in the Electronic Supplementary Material (Table S4, Fig. S3 and Table S5).

#### Dissolved fraction determination of different ENP suspensions

Based on the results of the method comparison, the dissolved fraction of the other ENP suspensions included in this study was determined by means of ultrafiltration, as described above. Again, for method validation, ICP single-element standards of the respective elements as well as blank samples were included in the analyses. Except for ZnO, the dissolved fractions of the WS were stable over the experimental time of approximately 2 weeks. In case of ZnO, freshly diluted suspensions were compared to a WS diluted 24 h prior to the experiment.

## Results and discussion

### General characterization of the ENP suspensions

#### Particle size distribution and zeta potential

Particle size distributions of the ENP suspensions as well as the zeta potentials are summarized in Table 1. Beside the mean values of the hydrodynamic diameter and the zeta potential, the peak width (DLS, zeta potential) and the standard deviation (NTA) given by the software are shown. For DLS the *z*-averages of the measurements are given. For TEM analyses, the standard deviation of the particles measured is provided. Representative TEM images of the ENP suspensions can be found in the Electronic Supplementary Material (Fig. S1).

**Table 1:** Particle size distribution and zeta potential of the ENP suspensions. Beside the mean values, the standard deviations (NTA, TEM) or the peak width (DLS, Zeta) are given respectively. Analyses of DLS, NTA, and zeta potentials were conducted in (at least) five replicates. If possible, the particle sizes were additionally estimated on the basis of TEM images (*n* ≥ 57); due to aggregation, the size of TiO_2_ and CeO_2_ of individual particles were not measurable

	Ag	TiO_2_	CeO_2_	ZnO	Au 10 nm	Au 200 nm
DLS	52 ± 20	108 ± 2	24 ± 9	429 ± 105	29 ± 12	181 ± 87
NTA	55 ± 19	197 ± 72	86 ± 4	285 ± 106	(too small)	247 ± 10
TEM	18 ± 4	(<20 nm)	(<20 nm)	(13 ± 5)	9 ± 2	279 ± 18
Zeta potential [mV]	−18 ± 7	−30 ± 7	40 ± 10	−32 ± 4	−42 ± 1	−46 ± 9

The difference of the particle sizes obtained by different methods is a well-known phenomenon since the properties of the suspensions (mainly particle shape, dispersity, agglomeration/aggregation state) can strongly influence the measurements [5, 10, 43]. Moreover, the values determined on the basis of the TEM images represent the size of the core particles, whereas by application of DLS and NTA measurements the (larger) hydrodynamic diameter is obtained (e.g., Ag or ZnO). In case of bigger particles such as Au 200 nm, the influence of the electric dipole layer surrounding the particle on the size measured is less pronounced and, hence, the results are more similar. For TiO_2_ and CeO_2_, the TEM images revealed that the particles are present as aggregates, leading to stable suspensions with much higher hydrodynamic diameters. Also in case of ZnO, agglomerates were observed. The instability was confirmed by a broad size distribution determined by DLS and NTA as well as by the time-dependant agglomeration observed (already mentioned in the experimental section). Nevertheless, for all three suspensions, the TEM images indicate that not exclusively aggregated but also single particles were present. Beside this, the particles of the TiO_2_, CeO_2_, and ZnO suspensions were not spherical, which also biases the results determined by light-scattering methods such as DLS and NTA [5, 10, 43]. Regarding the zeta potentials, with the exception of Ag, all values measured were |>28 mV|, indicating repulsive forces between the particles that dominate the stability with increasing values of the zeta potential [44–46]. With the exception of CeO_2_, all samples showed negative values. The lower value of Ag may explain the polydispersity of the suspension because the more intense attractive forces between the particles can cause aggregate formation.

### Total concentration

#### Sample preparation procedures

**Fig. 1:**
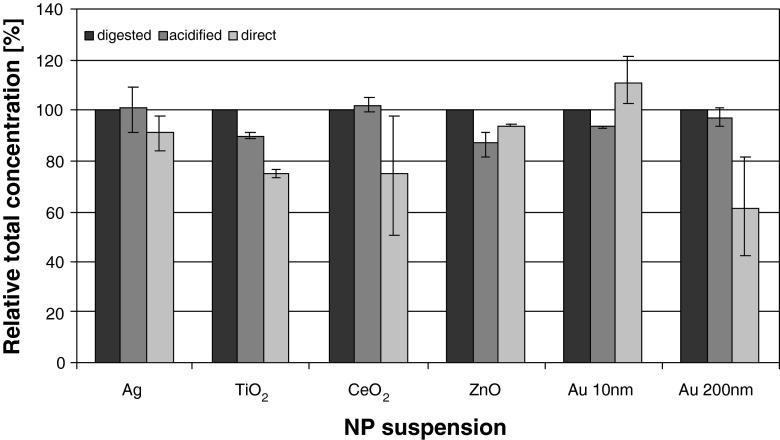
Relative concentration of ENP WS determined by means of ICP-QMS after microwave assisted digestion (*anthracite*), acidification (*gray*), and direct application (*light gray*). For each suspension, ≥6 replicates were analyzed. Results are presented as percentage of the results obtained upon microwave digestion (set as 100 %). *Error bars* represent the minimum and maximum values (acidification, direct application) in relation to the values of the digested samples

Figure 1 exhibits the relative total concentration obtained upon different sample preparation procedures (microwave digestion, acidification, as well as direct application). The values achieved after microwave digestion were set as 100 %. Error bars indicate the relative minimum and maximum offset values (acidification, direct application) in reference to the microwave digestion.

Regarding the acidified samples, the relative concentrations of Ag (91.0–108.6 %, mean 100.6 %), CeO_2_ (99.0–105.3 %, mean 102.2 %), and Au 200 nm (94.1–100.7 %, mean 96.5 %) were similar to those achieved upon microwave-assisted digestion (bias <5 %), even though for gold a slightly lower mean value was determined. A reduction of relative concentrations by more than 5 % was found for TiO_2_ (88.5–91.1 %, mean 89.8 %), ZnO (81.0–91.5 %, mean 87.4 %), and Au 10 nm (92.6–94.2 %, mean 93.6 %). The concentrations of the aqueous suspensions determined via direct ICP-QMS analysis without further sample pretreatment were, except for Au 10 nm (102.5–121.5 %, mean 111.2 %) and ZnO (93.3–94.2 %, mean 93.7 %), lower than those after acidification: Ag (84.0–97.7 %, mean 90.8 %), TiO_2_ (73.3–76.5 %, mean 74.9 %), CeO_2_ (50.3–98.2 %, mean 74.8 %), and Au 200 nm (42.6–82.1 %, mean 61.1 %). These findings indicate either losses during the transport of the particles into the plasma via tubings, nebulizer, and spray chamber or an incomplete atomization/ionization of the particles. The improved comparability of the acidified Ag, TiO_2_, CeO_2_, and Au 200 nm suspensions to the digested samples is possibly caused by a partial dissolution and/or reduction of the particle sizes, which, in turn, may have reduced the biases caused by insufficient transport or atomization/ionization processes. This effect is in accordance to several studies which indicate that the solubility of nanoparticles increases at low pH values [16, 24, 27, 28] or that particles may dissolve completely after acidification [28, 47]. However, for ZnO and Au 10 nm, inverse effects were observed which might be the result of agglomeration/aggregation and sedimentation processes induced by the reduced pH value. An acidification can cause a shift of the zeta potential towards zero (point of zero charge) where the electrostatic repulsion forces are reduced and the van-der-Waals forces became more dominant causing agglomeration and destabilization of the suspension [46, 48, 49]. This may explain the results of the ZnO suspension, but probably not of the sterically (by surfactants) stabilized Au ENP suspensions, where a pH shift is expected to have lower impacts on the stability of the suspension [45, 46, 49, 50]. Additionally, assuming an equal composition of the matrix of the two Au ENP suspensions, a destabilizing effect should have been observed in both cases. Nevertheless, regarding the much higher particle concentration of the Au 10 nm suspension in comparison to the Au 200 nm suspension (5.9 × 10^12^ part/mL vs. 1.9 × 10^9^ part/mL; see Electronic Supplementary Material, Table S1), the probability of collision and adhesion of particles (particle-particle interaction) and, hence, also of agglomeration is increased. However, since the experiments were repeated three times by two different persons, each including three replicates of the Au ENP suspension, it is unlikely that the differences observed were caused by random errors (due to, e.g., incorrect handling, measurement settings, or contaminations). Since the concentrations of the digested samples were additionally verified by means of GF-AAS, only particle-related effects might have biased the ICP-MS measurements. The results of the Au 200 nm ENP suspensions, analyzed in parallel, indicate that the effect is, as mentioned above, somehow related to the particle size, rather than to particle effects in general. For a conclusive explanation of the phenomena, the effects of each step of the preparation procedure should thus be further investigated, including important parameters like the zeta potential or the agglomeration status of the suspensions, which was not the focus of this study.

For most metal-based nanoparticle suspensions, a microwave-assisted digestion is advised prior to ICP-QMS measurements to ensure a correct quantification of the total metal concentration. Nevertheless, the results also demonstrate that an acidification of the suspensions provides an alternative if a digestion procedure is not feasible (e.g., due to time limitations in test protocols or with regard to coupled techniques). In cases where the differences between microwave-assisted digestion and an acidification are negligible, the latter is preferable due to reduced source of errors during extensive preparation procedures (e.g., potential analyte losses). The results of the Au 10 nm samples show that for some ENP suspensions also a direct application may be possible which emphasizes that an ENP/matrix-matched preparation has to be thoroughly adapted.

#### Comparison of ICP-MS analysis and gravimetry

As an absolute method, a gravimetric approach was compared to the results of the ENP stock suspensions obtained by means of ICP-QMS after microwave-assisted digestion (Table 2).

**Table 2:** Comparison of concentrations and the respective confidence interval (CI; *α* = 0.05) determined gravimetrically (*n* ≥ 5) and by means of ICP-QMS after microwave assisted digestion (*n* ≥ 3) of the ENP stock suspension (SS)

	Ag [g/L]	TiO_2_ [g/L]	CeO_2_ [g/L]	ZnO [mg/L]	Au 10 nm [mg/L]	Au 200 nm [mg/L]
Gravimetric	97.1 ± 1.2	5.3 ± 0.2	210 ± 13	684 ± 66	474 ± 143	545 ± 130
Digestion SS	101 ± 11	4.6 ± 0.2	267 ± 4.9	293 ± 21	56.4 ± 2.2	72.5 ± 3.6

According to the results of the two approaches, the ENP suspensions analyzed can be divided into two groups. For the highly concentrated and surfactant free suspensions, namely Ag, TiO_2_, and CeO_2_, the concentrations determined by means of the two approaches were comparable with each other, whereas in case of the less-concentrated ZnO and Au ENP suspensions, which are stabilized in sodium citrate solution, remarkable differences were found. Since the concentrations of the ICP-QMS analysis were similar to the results of the previously described experiments (characterization of the WS; data not shown), it was suspected that the gravimetric approach is, in case of the ZnO and Au ENP suspensions, biased by residues of the matrix (sodium citrate and surfactants) remaining in the aluminum cups after the combustion process. Equally, in some TEM images (see Au 200 nm; Electronic Supplementary Material, Fig. S1), crystals were observed, presumably indicating remaining residues of the matrix. To further validate the results of the ICP-QMS measurements, the samples of the digested Au stock suspensions were additionally analyzed by means of GF-AAS, leading to similar results as determined via ICP-QMS: Au 10 nm 52.3 ± 1.2 mg/L, Au 200 nm 68.5 ± 1.4 mg/L. It can be concluded that a gravimetric approach is only valid for suspensions in an exactly known matrix without interfering components (like surfactants or stabilizers). Beside this, the uncertainties of the methods increase with a decreasing concentration of the suspension, since the determination of low weights leads to elevated measurement uncertainties. In case of the highly concentrated suspensions and/or big particles (Au 200 nm), it has to be considered that pipetting errors (by, e.g., remaining suspension in the pipette tip) bias the results, which explains the differences found in case of Ag, TiO_2_, and CeO_2_. Hence, especially in case of such samples, a high number of replicates is recommended to limit the statistical uncertainty. For Ag, TiO_2_, and CeO_2_, similar results were found with regard to the concentrations provided by the manufacturer (Electronic Supplementary Material, Table S1 and Table S2). In case of Au (especially Au 200 nm), the elemental concentrations determined were below those estimated on the basis of the concentrations given in particles/milliliter. This may be due to uncertainties caused by slight variations of the particle size distribution, which can lead to discrepancies to the calculated values (refer to Electronic Supplementary Material, Table S1 and Table S2). In contrast to that, the concentration of ZnO was approximately three times higher than expected from the producer information given.

#### Precision of ICP-MS measurements

**Fig. 2:**
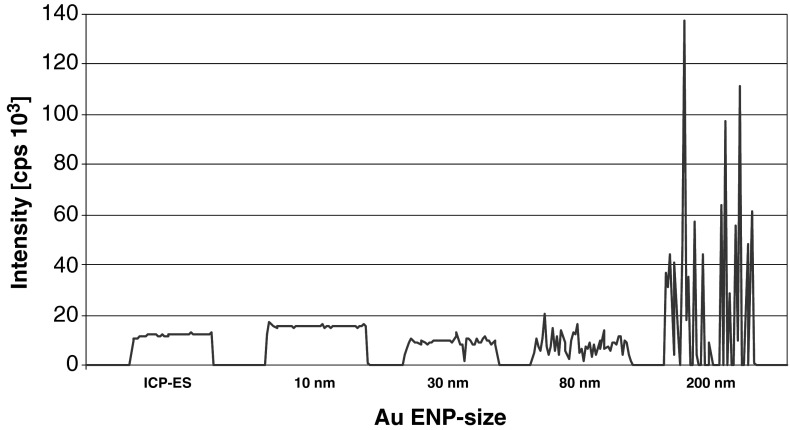
Direct application of Au ENP suspensions of different sizes (10, 30, 80, 200 nm) compared to a dissolved ICP-single element standard

The relative standard deviations (RSD%) of the ICP-QMS measurements of different sample preparation procedures were analyzed to test if particles, which are directly introduced into the ICP-MS system, cause signal instabilities in steady state measurements. The RSDs reflect the stability of the measurements and, thus, the precision of the ICP-QMS analyses (Table 3). To investigate, moreover, the size dependence of the signal stability, gold-ENP suspensions containing particles of different sizes (10, 30, 80, 200 nm) as well as a dissolved gold ICP-MS single element standard were applied to ICP-QMS. Figure 2 illustrates the signals obtained.

**Table 3:** Precision of the ICP-QMS analysis represented by the mean values of the relative standard deviations (RSD%) of the samples measured (five replicates) subsequent to different sample preparation procedures

	Ag (%)	TiO_2_ (%)	CeO_2_ (%)	ZnO (%)	Au 10 nm (%)	Au 200 nm (%)
Digested WS	2.2	3.2	1.1	1.9	1.3	2.1
Acidified WS	2.2	5.0	1.4	1.8	0.9	1.6
Direct WS	2.7	13.3	4.4	3.0	3.8	32.6

Except for silver, all ENP suspensions, which were directly introduced into the ICP-MS system, showed elevated RSDs, compared to the samples digested and/or acidified. Even though in case of CeO_2_, ZnO, and Au 10 nm, only slight differences in signal stability were observed. The ICP-QMS signal stability of directly introduced TiO_2_ and, especially, Au 200 nm suspensions were enhanced significantly (RSDs > 10 %). The results of the Au ENP suspensions of different particle size distributions illustrates that the stability of the signal decreased with increasing particle sizes (Fig. 2). No differences were found between the dissolved ICP-element standard and the Au 10 nm particles. In addition to the size of the individual particles, the aggregation state (in case of TiO_2_, CeO_2_, and ZnO) of the suspensions as well as the solubility of the materials probably influenced the measurements, which might explain the constant RSDs of the Ag ENP suspension, known to dissolve fast [20, 25, 26, 28]. In general, the effects observed are well known and used in SP-ICP-MS, where the concentrations and particle sizes of ENP suspensions are measured on the basis of signals of single particle events [34, 35]. However, in comparison to SP-ICP-MS, in classical ICP-MS measurements higher concentrations and longer dwell times are applied causing an overlay of several, not defined single particle events and an instable steady state signal (illustrated in Fig. 2). Moreover, the example of the Ag ENP suspension, which show only a marginally increased RSDs (refer to Table 3), demonstrates that this is (depending on the size, stability, and concentration of the suspension) not necessarily true for all kind of ENP suspensions.

Summing up, the results highlight that prior to any classical ICP-MS analysis of inorganic nanoparticle suspensions, it has to be investigated if and which kind of sample preparation procedure is required to ensure a correct and valid quantification of the (metal) constituents. Mostly, a microwave-assisted digestion seems to be the favorable practice, but the results of the Ag, CeO_2_, and Au 200 nm suspensions indicate that in some cases acidification is also suitable. Even though for small particles (like Au 10 nm) the results of a direct application are comparable to digested samples, this approach cannot be advised since instabilities of measurements are likely.

### Determination of the dissolved fraction

#### Comparison of different off-line fractionation approaches

**Fig. 3:**
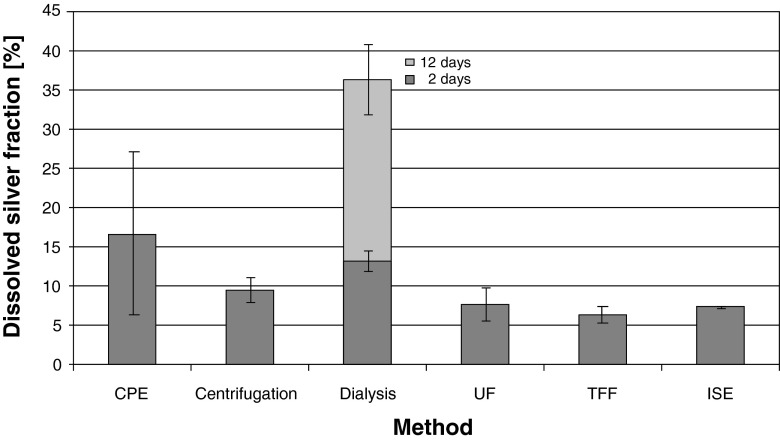
Dissolved fraction of silver ENP WS determined by means of six different methods applied (*n* ≥ 6). Results are presented as percentage of the total Ag concentration of the WS determined after microwave assisted digestion. *Error bars* represent the CI (*α* = 0.05). Except for ISE, concentrations were determined by means of ICP-QMS (for dilutions and measurement settings refer to the experimental section and Table S2 in the Electronic Supplementary Material). As a time-dependent method, for dialysis, the result obtained after 2 (*gray*) and 12 days (*light gray*) are shown. Filtration methods (UF, dialysis, TFF) were performed using membranes with a MWCO of 10 kDa

Testing different approaches to quantify the dissolved fraction of a Ag ENP suspension, five off-line fractionation methods as well as measurements with an ISE were compared; the results (mean value ± CI; *α* = 0.05) are presented in Fig. 3. Only for dialysis, the results after 2 (gray bar) and after 12 days (light gray bar) are presented, since during dialysis a continuous dissolution of silver was observed (see Fig. S2 in the Electronic Supplementary Material). In most cases, the results of the simultaneously analyzed dissolved ICP-element standard, included to test for the recoveries of the methods, were within a range of 100.0 ± 5.0 % (mean value ± CI; *α* = 0.05: centrifugation, 102.2 ± 11.1 %; dialysis, 99.7 ± 8.0 %; ultrafiltration, 95.8 ± 2.1 %; ion selective electrode, 102.1 ± 3.9 %). With the tangential flow filtration, recoveries of 87.5 ± 4.2 % were observed for the dissolved silver standard and 79.8 ± 42.2 % for the cloud point extraction. The silver concentrations of the blank samples, conducted with ultrapure water, were below the limit of detection (LoD, 0.26 μg/L; refer to Table S3, Electronic Supplementary Material).

The dissolved fractions determined via CPE (16.7 ± 6.5 %) and dialysis after 2 days (13.7 ± 1.5 %) were >10 %, whereas the results of the CPE showed the highest CIs of all methods applied. A slightly lower percentage for the dissolved fraction was determined by means of centrifugation (9.5 ± 1.6 %). Similar results were found after application of UF (7.7 ± 1.3 %), TFF (6.3 ± 1.2 %), and ISE (7.0 ± 0.3 %). In case of the CPE, the relatively high concentration of dissolved silver determined is most likely caused by particles remaining in the supernatant. As mentioned before, the method rather aims to extract and concentrate nanoparticles than to allow for quantitative extraction of dissolved silver [31, 40, 51]. However, depending on the properties of the nanoparticle suspensions, the respective matrix, and the parameters of the procedure (e.g., ENP concentration, pH, salinity), the recoveries determined in different studies ranged, mostly, from ∼65 % up to ∼110 % of the initial ENP concentration [31, 40, 51]. Hence, the variation found in these studies is comparable to the results presented here. This indicates that a certain amount of the particles is usually not incorporated in the TRX-114-phase and might bias the dissolved silver quantification. Apart from the low recoveries of the ICP-element standard, high CI values were obtained for both types of samples (ENP suspensions and element standard), indicating that the dissolved fraction was not precisely quantified. Nevertheless, since the approach can in principle be applied to different ENPs (beside Ag, e.g., also Zn, Au, or TiO_2_) in different matrices [31, 40, 41, 52, 53], a further methodological adaptation of the parameters may potentially enable also an accurate and precise determination of the dissolved fraction. Especially with regard to low concentration of ENPs in complex matrices, this approach may provide a possibility to quantify the concentration of the nanoparticles and the dissolved fraction simultaneously. Regarding the centrifugation, the dissolved fraction determined was slightly enhanced in comparison to the results of UF, TFF, and ISE, probably indicating a bias by remaining, or rather re-dispersed, particles in the supernatant. Even though in some samples a few particles (approximately 25–50/mL supernatant) were detectable by means of NTA, the number was too small for precise particle number determination. However, the advantage of this approach is that, in contrast to the other methods, the sample matrix remains unchanged (contrary to CPE or ISE) and, furthermore, that interactions with membranes (possible during dialysis, TFF or UF) can be excluded. Nevertheless, to ensure a complete sedimentation especially of small particles of a low density, prolonged centrifugation times of (possibly) several days and/or higher speed are required which, in turn, demands an adequate equipment allowing for ultra high speeds (fixed angle rotor and respective tubes) which causes higher costs. Moreover, the sedimentation process also depends on shape and surface coatings of the particles as well as the heterogeneity of the sample [54, 55]. In case of small particles (<20 nm), the influence of surface coating on the particle size and density becomes more dominant, which, in turn, influences the sedimentation coefficient and the centrifugation procedure [54]. Therefore, an adaptation of the method to the different characteristics of the respective ENP suspension (particle size, shape, polydispersity, matrix, etc.) is indispensable. Beside this, a thorough handling of the samples is crucial to avoid redispersion, which causes user dependence and may result in incorrect results. In case of dialysis, the ongoing release of silver (refer to Fig. S2 in the Electronic Supplementary Material) demonstrates that the method cannot be applied to determine the dissolved fraction of a given Ag ENP suspension because the primary ratio between particles and water is remarkably changed. Since the release of silver ions depends, among others, on the concentration [25, 26], dialysis is suitable to investigate the time-dependant dissolution kinetics [16, 26] but not to quantify the dissolved fraction of a given ENP suspension. In contrast to dialysis, centrifugal UF enables the determination of the dissolved fraction without matrix modifications. Possible interactions of silver ions with the membranes are, with regard to the recoveries of >95 % of the ICP-element standard, negligible, but have to be in general taken into account. Similar, clogging of the membranes by nanoparticles, tested by application of an ICP-element standard upon the filtration of ENP suspensions (recovery 108.1 % ± 3.0 %) can, in this case, be excluded. Moreover, UF is a fast and easily applicable method which is already commonly used to determine the dissolved fraction of ENP suspensions [21, 22, 24, 25, 30, 56]. In comparison to that, the results obtained by application of the tangential flow filtration are in good agreement with those obtained by the UF and ISE approach and show apparently good reproducibility. Nevertheless, several drawbacks became apparent during application: in some cases the liquid flow through the membranes was hindered, probably due to clogging of the pores, the approach is time-consuming and the recoveries of the ICP-element standard indicate losses during the procedure. Beside this, the hollow fibers were, at a price of ∼80 € per piece, the most expensive devices used during the study (compared to: Float-A-Lyzer for dialysis and Amicon ultra ultrafiltration units each ∼11 € per piece). In contrast to the other methods applied, the ion selective electrode provides a possibility to determine the concentration of silver ions in a sample and, thus, to obtain results not biased by nanoparticles. This was verified by standard addition of dissolved Ag ICP-element standard to NP suspensions (101.5 % ± 6.2 %) after calibration of the electrode. Hence, this approach can be taken as a control for the results of the other methods. However, although ISE facilitates the possibility for the fast and direct determination of the concentration of dissolved silver in different matrices [31, 40, 57], it is a tool only applicable to few ion-releasing ENPs (beside for Ag, ISEs are available for, e.g., Cd, Pb, and Cu) and, in comparison to ICP-MS measurements, to elevated dissolved silver concentrations (∼≥10 μg/L; information given by the manufacturer).

#### Dissolved fraction determination of different ENP suspensions

Based on the results achieved upon the method comparison conducted using a Ag ENP suspension, ultrafiltration was identified as best practice for the determination of the dissolved fraction of the other ENP suspensions used within the comparison of the sample preparation procedures. The recoveries of the ICP-element standard were in the range of 100.0 ± 5.0 % (mean value ± CI; *α* = 0.05: Ag, 95.8 ± 2.1 %; TiO_2_, 102.6 ± 4.6 %; CeO_2_, 99.3 ± 0.5 %; ZnO, 104.8 ± 6.4 %; Au 10 nm and Au 200 nm, 97.5 ± 0.01). Background concentrations for the elements analyzed (determined by filtration of ultrapure water) were, with the exception of Zn (0.4 ± 0.8 μg/L), below the respective limit of detection (refer to Table S3 in the Electronic Supplementary Material). The highest values for the dissolved fraction were determined for the suspensions of Ag (7.7 ± 1.3 %) and ZnO (4.4 ± 0.3 %). For ZnO, the dissolved fractions were determined for freshly diluted WS, whereas the results of silver are referred to the suspension used within the other experiments (described above). For ZnO, the dissolved fractions were additionally determined 24 h after the dilution. They showed increased values of 26.2 ± 8.0 % (*n* = 3). For CeO_2_, 0.4 ± 0.1 % was found; for TiO_2_ and the two Au suspensions, the concentrations within the filtrates were below the respective limits of detection (refer to Table S3 in the Electronic Supplementary Material). For these suspensions, no time-dependent dissolution was observed. The release of ions from Ag and ZnO ENPs and the dependence of this process on several parameters (e.g., concentration, pH value, particle size, aggregation state, additives) [16, 21, 22, 25, 26, 28]) is a well-known phenomenon, especially discussed in relation to the toxicological impacts of these nanoparticles [15, 16, 26, 30, 56, 58–61]. In case of CeO_2_, the solubility in aqueous matrices is expected to be low, even if at acidic pH values (pH <4) a certain dissolution is possible [24, 62]. However, since the samples were not acidified and the TEM images (refer to Fig. S1 in the Electronic Supplementary Material) of CeO_2_ indicate the presence of very small individual particles, the results of the dissolved fraction were probably biased by particles that have passed the pores of the membranes. Similar, the solubility of TiO_2_ ENPs can be slightly increased at low pH values but is generally expected to be very low in water [27, 29]. Likewise, the release of soluble gold clusters from Au ENPs is, without the attachment of special ligands, improbable in water [63–65]. Hence, the dissolved fractions determined are in good agreement with the results that can be expected from other studies.

Taken together, the results of the comparison of methods for (quantitative) off-line fractionation and ISE to determine the dissolved fraction of different ENP suspensions highlight that the suitability of the methods applied have to be thoroughly verified for each type of metal-based nanoparticle in a given matrix. Beside the advantages and limitations of different methods, the parameters influencing the properties of the nanoparticles (e.g., size, aggregation state, solubility, zeta potential) and/or of the matrix (e.g., pH value, temperature, ionic strength) have to be considered carefully [22, 56, 66]. Ultrafiltration is a fast and easily applicable method which is suitable to a variety of different ENP suspensions and it provides the possibility to obtain results comparable between different working groups from different scientific disciplines. Nevertheless, in case of more complex matrices (e.g., environmental samples or media used in (eco)toxicological test systems or food), the suitability of the method has to be verified to avoid biases (by e.g., interactions of ENPs or the matrix with the membranes).

## Summary and conclusions

Focused on daily routine and ICP-MS analysis of metal-based nanoparticles, different strategies for (1) the determination of the total metal concentration as well as (2) the dissolved metal fraction were compared. (1) It has been shown that a direct application of nanoparticle suspensions to an ICP-MS system does, applying steady state analyses, mostly not provide reliable data for total metal concentrations.

In fact, without any further sample preparation, it is very likely that imprecise results and/or instabilities of the measurements occur. Even though for some ENP suspensions (in this study Ag, CeO_2_ or Au 200 nm) acidification was identified as an optimal practice, microwave-assisted digestion can be taken as a universally reliable method. In cases where an (extensive) sample preparation is not feasible (e.g., direct coupling of analytical devices to ICP-MS or due to time limitations in extensive test protocols), the uncertainties in the respective matrix should and can be easily addressed prior to an experiment. (2) Regarding the determination of the dissolved fraction, six methods were compared by application to an Ag ENP suspension to identify the most suitable approach. It has been shown that, in principle, several methods can be applied.

Nevertheless, problems like time-dependent dissolution (dialysis), methodological and handling difficulties (CPE, centrifugation, TFF), or elevated costs (TFF) have to be considered. As a method suitable for different ENP suspensions, centrifugal ultrafiltration provides an easy to handle and moderately expensive tool for the separation of the dissolved fraction from the particles. Moreover, by application of this approach to the ENP suspensions included in this study, the general suitability of the method for a variety of different nanomaterials was shown.

At the same time, the results also demonstrate that even for presumable simple analytical tasks concerning quantification and characterization of ENP suspensions, the analytical tools available are not necessarily suitable for all nanomaterials. It hence remains still necessary to verify the applicability of standard protocols for a given experimental approach and the ENP suspensions analyzed. Nevertheless, it has been shown that it is possible to identify and validate easily implementable standard procedures, which are crucial to ensure the comparability of the results of different laboratories and to provide a basis for the development and implementation of regulatory frameworks.

## Electronic supplementary material

Below is the link to the electronic supplementary material.

ESM 1 (PDF 581 KB)
