# Supplementary material for: ICP-MS-based characterization of inorganic nanoparticles—sample preparation and off-line fractionation strategies
Source: Anal Bioanal Chem. 2013 Dec 1;406(2):467–79. doi: 10.1007/s00216-013-7480-2 (PMC3885803; doi:10.1007/s00216-013-7480-2)
Supplement: Supplementary file 1 — (PDF 581 KB) [file 216_2013_7480_MOESM1_ESM.pdf]

Analytical and Bioanalytical Chemistry

Electronic Supplementary Material

ICP-MS-based characterization of inorganic nanoparticles—sample preparation and off-line fractionation strategies

Anne-Lena Fabricius, Lars Duester, Björn Meermann, and Thomas A. Ternes

Experimental

Table S1. Information about the nanoparticle suspensions used given by the manufacturer

	**AgPURE-W**	**TiO_2**	**CeO_2**	**Au 10 nm**	**Au 200 nm**	**ZnO**
	ras materials GmbH, Germany	Laboratory synthesized	NYACOL; Nanotechnolo-gies, Inc, USA	Sigma-Aldrich, Germany		Particular GmbH, Germany
Concentration	10.0 ± 0.50 %	5.9 g/L	20 wt.%	$5.9 \cdot 10^{12}$ part/mL	$1.9 \cdot 10^{9}$ part/mL	100 mg/L
Additives	Ammonium nitrate (3-15%)	None	Nitrate (0.2 mol/mol)	Sodium citrate buffer (100 mg/L) + proprietary stabilizing surfactant		Sodium citrate buffer (100 mg/L)
Core size; (diameter) [nm]	15 (99%< 20)	100	10-20	8-12	180-220	-
Hydrodynamic diameter [nm]	-	-	-	18-30	175-235	136.1 ± 4.2

Table S2. Details about microwave assisted digestion procedures

	Ag	TiO_2	CeO_2	ZnO	Au	
HNO_3 (65%)	1.4 mL	-	1.2 mL	0.35 mL	0.35 mL	
H_2SO_4 (96%)	-	1.4 mL	-	-	-	
HCl (30%)	-	-	-	1.05 mL	1.05 mL	
H_2O_2 (30%)	-	-	0.2 mL	-	-	
Vessel material	Teflon	Teflon	Teflon	Teflon	Glass	
Internal Standard	Ru (1 mg/l) 0.1 mL					
Sample	0.5 mL					
Dilution	1:100.000	1:1.000	1:100.000	1:100	1:100	
Assumed concentration	~1 mg/L	~5.9 mg/L	~2 mg/L	~1 mg/L	99.4 µg/L*	253 µg/L*
pH value	5.8	5.8	4.5	5.9	6.2	6.3
Reference material	TMDA52.3**: 20.6 ± 1.8 µg/L	TMDA52.3**: 120.0 ± 7.6 µg/L	SPS-SW2*** 2.5 ± 0.02 µg/L	TMDA**: 263.0 ± 25.3 µg/L	Noble metals****: 100 µg/L	
ICP-element std	Ag (1 mg/L)	Ti (1 mg/L)	Ce (1 mg/L)	Zn (1 mg/L)	Au (1 mg/L)	

* Conversion from concentrations given in particles/mL (information of the customer) to the concentration of Au in µg/L were conducted in accordance to [1].

** Certified Reference Waters for Trace Elements, Environment Canada.

*** Surface water – trace metals, Spectrapure Standards, Norway.

**** VHG certified NIST-traceable multi element aqueous standard, VHG Labs, Inc (LGC standards), USA.

In case of Ag, TiO_2 and CeO_2 ENP suspensions the digestion procedure was carried out in Teflon vessels. For the Au ENP suspensions glass vials were used to avoid possible interaction with the Teflon surfaces. In case of ZnO, after testing both materials, also Teflon vials were chosen due to lower blank values and better recoveries. For ICP-MS measurements digested samples were transferred to polypropylene centrifuge tubes (VWR, Germany) or, in case of Au, to Rotilabo®-screw neck ND24 glass vials (Carl Roth, Germany).

Table S3. Parameters of ICP-QMS analysis

Isotopes of the elements analyzed and the respective limits of detection (LoD; blank + 3 sigma) and limits of quantification (LoQ; blank + 10 sigma; acidic and aqueous matrices). Collision cell modus, [He], was conducted at a helium gas flow of 3 ml/min. Certified concentrations of the reference materials (CRM) used for method validation are given for each element.

	**Ag** [µg/L]		**Ti** [µg/L]		**Ce** [µg/L]		**Zn** [µg/L]		**Au** [µg/L]	
Isotope	^{107}Ag		^{47}Ti [He]		^{140}Ce		^{66}Zn [He]		^{197}Au	
Matrix	acid	water	acid	water	acid	water	acid	water	acid	water
LoD	0.26	0.21	0.71	0.18	0.21	0.21	1.0	0.1	0.35	-
LoQ	0.67	0.61	1.73	0.49	0.64	0.64	2.29	0.18	0.93	-
CRM	TMDA 52.3		TMDA 52.3		SPS-SW2		TMDA 52.3		Noble metals	
			TM 27.3**: 2.01 ± 0.26 µg/L				TM 27.3**: 16.2 ± 2.6 µg/L			

** Certified Reference Waters for Trace Elements, Environment Canada (see Table S2).

Results and discussion

Fig. S1. TEM-images of nanoparticles suspensions

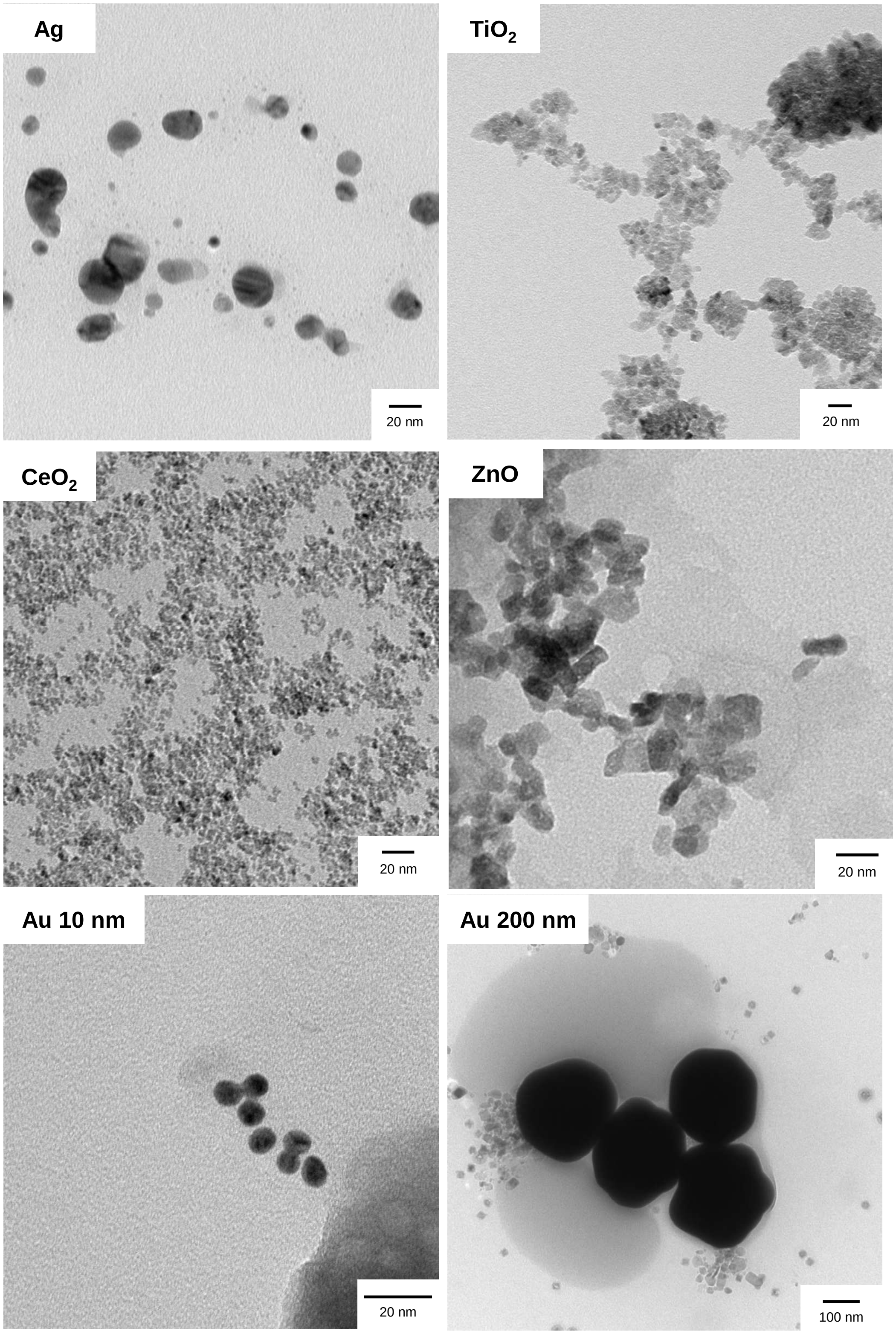

Centrifugation: calculation of the run time

The time needed for sedimentation of the silver nanoparticles was calculated in accordance to Griffith 2010 [2] and the information given in the users manual of the rotor (TH-641, Thermo Scientific, Germany). The sedimentation time was estimated as follows:

The time (T) required for sedimentation of a particle to the bottom of the tube is defined by the ratio of the clearing factor of the rotor (K) and the sedimentation coefficient (S) of the particles (Eq.1):

$$T = \frac{K}{S} \qquad \text{Eq.1}$$

The K-Factor describes the relative pelleting efficiency (sedimentation of particles to the bottom of the tube) of the rotor and can be calculated as followed (Eq.2):

$$K = (253{,}000)\left[\ln\left(\frac{r_{\max}}{r_{\min}}\right)\right] \div \left(\frac{rotor - speed}{1{,}000}\right)^2 \qquad \text{Eq.2}$$

The rotor speed was set to 41,000 rpm, the maximum and minimum distance between particle and the center of rotation (r_{max} and r_{min}) are determined by the rotor (TH-641, Thermo Scientific, Germany) and the length of the tubes:

r_{max} = 7.19 cm

r_{min} = 15.32 cm

The sedimentation coefficient was calculated in accordance to the following equations (Eq. 3 & Eq. 4):

$$S = \frac{v}{\omega^2 \cdot r} \qquad \text{Eq.3}$$

v = sedimentation velocity of the particle [cm/s]

ω = rotor speed in [rad/s]

$$\omega = \frac{2rpm}{60}; rpm = 41{,}000 \qquad rpm = 41{,}000$$

$$v = \frac{d^2(\rho_P - \rho_L)}{18 \cdot \mu} \cdot g \qquad \text{Eq.4}$$

d	=	diameter of the particle [cm]	
ρ_P	=	density of the particle	
ρ_L	=	density of the liquid	= 1 g/cm^3
µ	=	viscosity of the liquid medium	= 1.52 x 10^{-3} Pa s
g	=	gravitational force;	= 13.43 x 10^6 m/s^2

$$g = \omega^2 \cdot r$$

The time required for the sedimentation of a 50 nm Ag particle in a tube of approximately 8 cm was ~1.52 days (36.5 hours, 41,000 rpm). Since particle size distribution of the Ag ENP suspension showed a certain polydipersity (refer to results and discussion). Hence, the centrifugation time was prolonged to 48 hours to ensure the sedimentation of smaller particles. Moreover, only the upper ~1 cm from the surface of the liquid within the centrifugation tube was sampled.

Fig. S2. Time dependant dissolution during dialysis

Dissolution of Ag^{+} from Ag ENP suspension of three samples (diamond, square and triangle) over a period of 29 days.

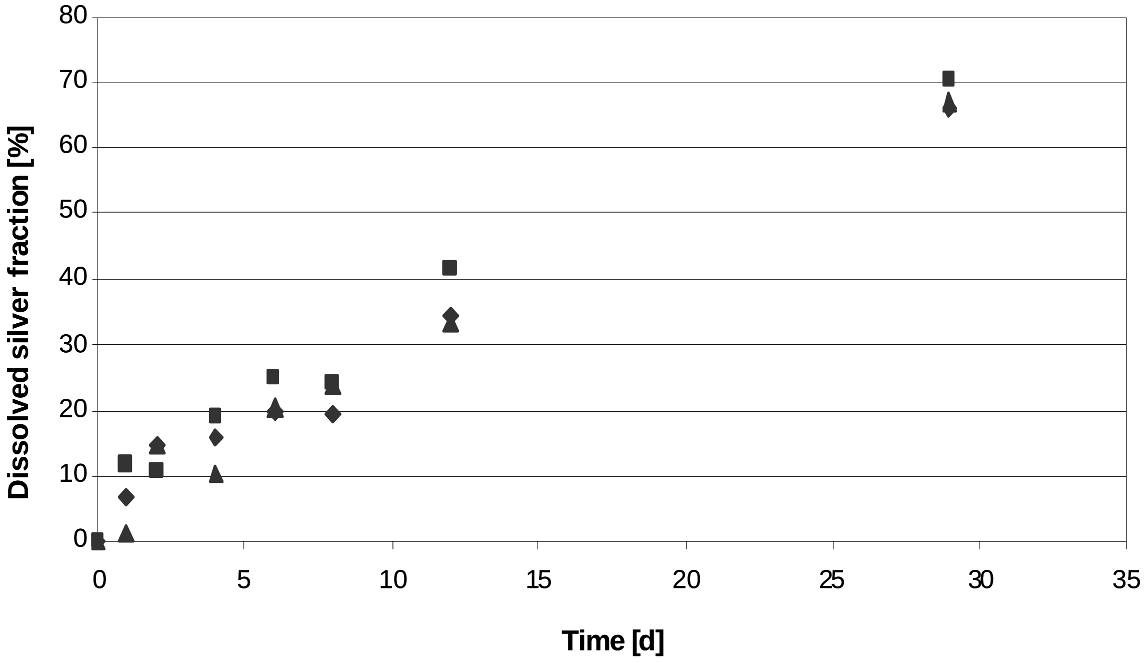

Calibration and results of the standard addition in ion selective electrode measurments

Table S4. Calibration of the silver ion selective electrode was conducted within a concentration range of 0.1 mg/L to 100 mg/L (values measured)

Concentration	log (Concentration)	Voltage [mV]
Ag [mg/l]	log(Ag)	U [mV]
0.1	-1.00	261
0.2	-0.70	266
0.5	-0.30	278
1	0.00	301
2	0.30	317
5	0.70	341
10	1.00	359
20	1.30	376
50	1.70	398
100	2.00	413

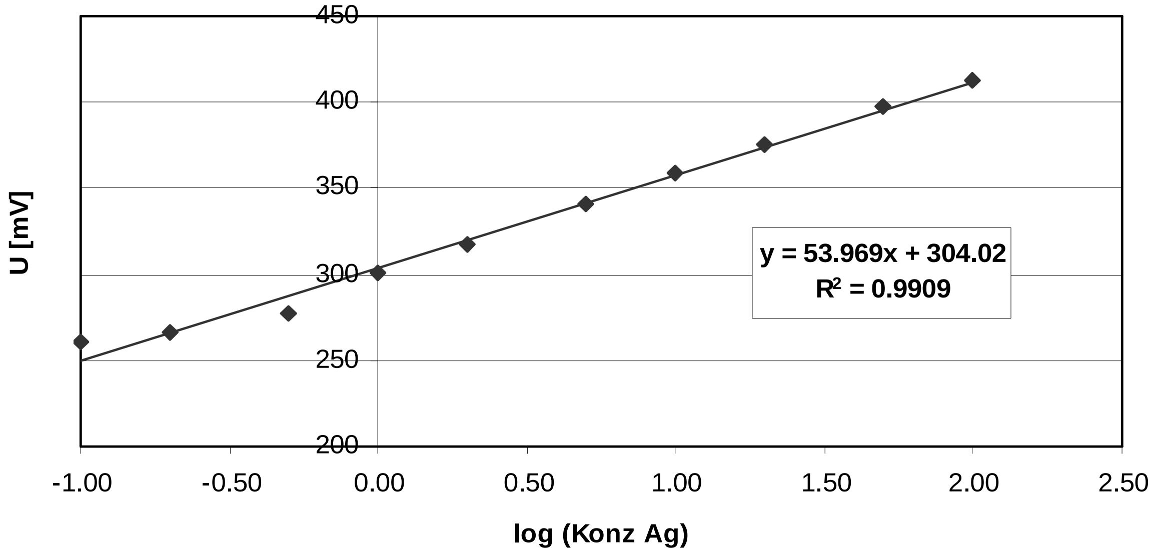

Fig. S3. Calibration curve of ISE

Table S5. Prior to further measurements, the response of the electrode was tested by a standard addition procedure. A Ag ENP working suspension (WS 1:10,000 dilution of the stock suspension) was spiked with a ICP element standard solution of different concentrations (see below). The concentration of dissolved silver determined by ISE was calculated on basis of the equation of the calibration curve. After blank correction, the recoveries were calculated on the basis of the concentrations of the Ag element standard added

	**Voltage**	**Concentration**	**Blank correction**	**Recovery**
	U [mV]	Ag^+ [mg/L]		[%]
WS + 0 mg/L Ag (blank)	272	0.26		
WS + 1 mg/L Ag	309	1.24	0.98	98.16%
WS + 2 mg/L Ag	321	2.06	1.81	90.43%
WS + 5 mg/L Ag	342	5.06	4.80	96.00%
WS + 10 mg/L Ag	359	10.44	10.19	101.86%
WS + 20 mg/L Ag	375	20.66	20.41	102.04%
WS + 50 mg/L Ag	396	50.62	50.36	100.73%

References

1. Lewis, D.J., et al., *Luminescent nanobeads: attachment of surface reactive Eu(III) complexes to gold nanoparticles.* Chemical Communications, 2006(13): p. 1433-1435.
2. Griffith, O.M., *Practical Techniques for Centrifugal Separations.* Thermo Fisher Scientific; Application guide, 2010.
